# Notochordal cells: A potential therapeutic option for intervertebral disc degeneration

**DOI:** 10.1111/cpr.13541

**Published:** 2023-09-11

**Authors:** Yanhu Li, Haijun Zhang, Daxue Zhu, Fengguang Yang, Zhaoheng Wang, Ziyan Wei, Zhili Yang, Jingwen Jia, Xuewen Kang

**Affiliations:** ^1^ Lanzhou University Second Hospital Lanzhou People's Republic of China; ^2^ Orthopaedics Key Laboratory of Gansu Province Lanzhou People's Republic of China; ^3^ The Second People's Hospital of Gansu Province Lanzhou People's Republic of China

## Abstract

Intervertebral disc degeneration (IDD) is a prevalent musculoskeletal degenerative disorder worldwide, and ~40% of chronic low back pain cases are associated with IDD. Although the pathogenesis of IDD remains unclear, the reduction in nucleus pulposus cells (NPCs) and degradation of the extracellular matrix (ECM) are critical factors contributing to IDD. Notochordal cells (NCs), derived from the notochord, which rapidly degrades after birth and is eventually replaced by NPCs, play a crucial role in maintaining ECM homeostasis and preventing NPCs apoptosis. Current treatments for IDD only provide symptomatic relief, while lacking the ability to inhibit or reverse its progression. However, NCs and their secretions possess anti‐inflammatory properties and promote NPCs proliferation, leading to ECM formation. Therefore, in recent years, NCs therapy targeting the underlying cause of IDD has emerged as a novel treatment strategy. This article provides a comprehensive review of the latest research progress on NCs for IDD, covering their biological characteristics, specific markers, possible mechanisms involved in IDD and therapeutic effects. It also highlights significant future directions in this field to facilitate further exploration of the pathogenesis of IDD and the development of new therapies based on NCs strategies.

## Graphical abstract

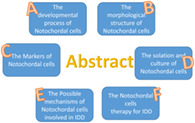

## 1 INTRODUCTION

The intervertebral disc (IVD) is a fibrocartilaginous joint structure situated between the vertebral bodies, comprising of nucleus pulposus (NP), annulus fibrosus (AF) and upper and lower cartilage endplates (CEP), facilitating flexion, extension and rotational movements of the spine.^1^ The NP tissue is composed of nucleus pulposus cells (NPCs) and their ECM, including glycosaminoglycans (GAGs) and type II collagen. These components maintain the hydration of the NP tissue through osmotic pressure provided by GAGs, which is essential for resisting axial pressure in the spine and plays a crucial role in intervertebral disc degeneration (IDD).^2^, ^3^ The AF cells are situated within a matrix abundant in type I collagen, which confers resistance to lateral expansion of the IVD during spinal loading.^4^ Anchoring the IVD to the upper and lower vertebrae is the CEP.^5^ As the largest non‐vascular organ in the body, nutrients are absorbed by cells residing in the NP through permeable CEP and AF.^6^ IDD is the primary aetiology of low back pain (LBP) in clinical practise, with ~40% of LBP cases being attributed to IDD.^7^, ^8^, ^9^ According to statistics, around 80% of adults will experience LBP at some point in their lives, which significantly impacts their quality of life and imposes a substantial economic burden on society.^10^

Currently, conventional treatments such as conservative physical therapy, block treatment and surgical intervention are the primary methods for managing LBP caused by IDD.^11^, ^12^ While surgical intervention may offer superior short‐term efficacy,^13^ it alters spinal biomechanics leading to further degeneration of adjacent IVDs and surrounding tissues, and even poses a risk of spinal cord injury.^14^ In the management of pain, prescription opioids are utilised by 30%–60% of patients with LBP, however, studies have not yet evaluated the long‐term efficacy, tolerability and safety of this medication.^15^, ^16^ At present, the current status of medical therapy for IDD is mainly to relieve symptoms with non‐steroidal anti‐inflammatory drugs and muscle relaxants, and there are no drugs that target the specific mechanism of the degeneration, because it is still not completely understood.^17^, ^18^ These treatments primarily aim to alleviate symptoms and reduce inflammation without targeting the pathogenesis of IDD.^19^ Thus, they cannot provide a fundamental cure for IDD.^20^, ^21^ The optimal treatment for IDD should aim to enhance the proliferation of IVD's NPCs and their ECM secretion, promote anabolic processes, inhibit catabolic processes and prevent ECM degradation by related enzymes in order to maintain IVD homeostasis.^22^ Bioremediation of IVD should ideally target the underlying degenerative process and promote regeneration of the degraded IVD, ultimately aiming to restore biomechanical function and tissue integrity to that of a healthy IVD, thereby ensuring spinal movement, flexibility and stability.^23^ Regenerative strategies for IDD primarily focus on the NP and AF, as they are responsible for most of the IVD's functions.^24^, ^25^, ^26^ In recent years, cell therapy has emerged as a research hotspot due to its potential to intervene in early‐stage IDD while preserving spinal mechanical characteristics. Although allogeneic mesenchymal stem cells (MSCs) have been utilised in numerous clinical trials for the treatment of IDD, resulting in improved function and pain relief among patients, sufficient evidence is needed to support the ability of this therapy to regenerate degraded IVDs.^27^

However, in recent years, there has been an increasing focus on the relatively neglected NCs within the IVD, with research indicating a potential correlation between NCs reduction or disappearance in adult NP tissue and IDD.^28^ While NCs naturally disappear after human adulthood, they persist for extended periods of time in certain vertebrates such as rabbits, cattle and dogs.^29^ During the maturation and senescence of the IVD, larger vacuolated NCs are replaced by smaller cartilage‐like NPCs. This coincides with the onset of IDD, indicating that NCs play a crucial role in maintaining IVD tissue health.^28^ NCs are an additional cellular component of the NP in vertebrate IVD, alongside cartilage‐like NPCs. They play a regulatory role in NPCs proliferation and promote ECM production through various growth factors, transcription factors (including transforming growth factor β [TGF‐β]) and signal transduction pathways.^30^

Furthermore, the released growth factors such as TGF‐β, collagen type II down‐regulating protein and proteoglycan up‐regulating protein by NCs exhibit anti‐apoptotic effects that impede NPCs apoptosis via inhibition of caspase‐9, caspase‐7 and caspase‐3 activities.^31^ Moreover, the administration of NCs can regulate the expression of matrix metalloproteinase‐3 (MMP‐3), aggrecan, type II collagen and recombinant tissue inhibitors of metalloproteinase 1 (TIMP1) genes to inhibit ECM degradation and maintain homeostasis in NP tissue.^32^ Additionally, NCs derived from rabbits have been found to induce differentiation of cyclooxygenase‐2 and TGF‐β, which can reduce inflammatory markers.^32^ In addition, Aguiar and Maldone do et al.^33^ postulated that NCs could serve as guidance cues for the migration of surrounding MSCs to IVD tissues and induce their differentiation into NPCs, thereby further regulating cell apoptosis and playing a crucial role in IVD tissue homeostasis.^34^

In this review, we first present the latest advancements in the developmental process of NCs, followed by a summary of their morphological structure and specific markers. We then provide an overview of isolation methods and culture conditions for NCs, before analysing their potential involvement in IDD and exploring therapeutic strategies based on NCs.

## 2 BIOLOGICAL CHARACTERISTICS OF NCS

### 2.1 The developmental process of NCs

Prior to delving into the developmental process of NCs, it is imperative to introduce the notochord, a hallmark of vertebrate body axis formation and an essential component of the embryonic axial skeleton.^35^, ^36^ The notochord undergoes development between the 15th and 30th day of human embryonic development, during which certain signalling molecules are secreted to induce the paraxial mesoderm to form cell clusters known as somites.^37^, ^38^ Each somite gives rise to sclerotome, dermatome and myotome tissues that eventually differentiate into axial bone, dermis and skeletal muscle.^39^, ^40^ As the embryo develops, the notochord is compressed by the formation of vertebrae into the region that will later form the IVD.^41^ In essence, the notochord is a rod‐like structure consisting of thick, vacuolated embryonic notochordal cells (eNCs) surrounded by a sheath composed of collagen, laminin, fibronectin and proteoglycans (primarily chondroitin sulphate and heparan sulphate).^42^

#### 2.1.1 The eNCs differentiate into NCs

The eNCs in zebrafish have been extensively researched, and each eNC produces a large intact vacuole that occupies the entire cell volume and is filled with fluid.^43^ The presence of this large hydrated vacuole, combined with the restriction of the notochordal sheath, determines the structural properties of the notochord, which only allows it to extend along its axis.^34^ Human eNCs are present as early as 3.5 weeks of gestation, but they later aggregate in IVD region between 8 and 10 weeks of gestation.^41^ During the 10th to 18th week post‐fertilisation stage, eNCs differentiate into NCs within the developing NP tissues.^41^ These NCs derived from eNCs retain vacuoles similar to those found in eNCs. However, these vacuoles become increasingly fragmented compared to the large and unfragmented ones observed in eNCs.

In comparison to eNCs, the developed NCs exhibited a greater capacity for ECM generation and secretion of abundant ECM enriched with GAGs, leading to the formation of cell clusters.^44^ Although the exact mechanism underlying the conversion of eNCs into NCs remains unclear, it is closely associated with the stress experienced by eNCs during vertebral development when they are compressed into the IVD region.^45^

#### 2.1.2 NCs differentiate into NPCs

With the further maturation of the embryo, human vacuolated NCs undergo differentiation into more mature, intracellular vacuole‐free, cartilage‐like NPCs at the 22nd week after fertilisation.^46^ The differentiation process of NCs into NPCs is a complex mechanism involving multiple factors including genetics. However, the specific differentiation pathway remains unclear and requires further investigation in future studies. The most prominent morphological and structural disparity between NPCs and NCs is the absence of vacuoles in NPCs, which is associated with the impact of osmotic pressure,^47^, ^48^, ^49^, ^50^ mechanical stress^51^, ^52^ and nutritional status.^53^ Consequently, these vacuole‐free NPCs would gradually differentiate into heterogeneous cell populations with their own phenotypic and functional characteristics.^54^, ^55^, ^56^

#### 2.1.3 Disappearance of NCs

Mature NPCs of sheep,^54^ goats^57^ and cattle^58^ have been demonstrated to lack NCs, whereas those of mice,^58^ rabbits,^58^ rats^58^ and pigs^54^ contain NCs and exhibit a lower incidence of IDD. These findings strongly suggest that the presence or absence of NCs is closely associated with IDD. In addition, Smolders et al.^59^ discovered that NCs in dogs with chondrodystrophy disappeared around 1 year of age, while those in non‐chondrodystrophic dogs could be maintained until middle and old age. The former group was more susceptible to IVD disease compared to the latter, providing further evidence for the hypothesis that reduction or disappearance of NCs may lead to IDD. However, it is unfortunate that human NCs begin to decline from the 22nd week after fertilisation,^46^ and will continue to decrease or even disappear with age after birth, ultimately being replaced by mesoderm‐derived cartilage‐like NPCs at around 10 years of age.^54^ The reduction of NCs leads to a decrease in the source of NPCs, resulting in insufficient replenishment of damaged or destroyed NPCs and subsequently compromising the anti‐injury ability of IDD. This phenomenon serves as an important pathological basis for IDD.^60^, ^61^ However, the specific mechanism underlying the decline or even disappearance of human NCs remains unclear and may be associated with cell apoptosis and differentiation towards terminal differentiation.

### 2.2 The morphological structure of NCs

The morphological structure of NCs varies among different species, and even between homozygous and heterozygous individuals within the same species. Hunter et al.^54^ demonstrated this by studying purebred and hybrid dogs at 2 years of age, finding that purebred dog NCs are relatively small and unconnected while those in hybrid dogs are larger and interconnected. In general, clusters of single or multiple cells containing NCs are distributed throughout the NP tissue. These cell clusters are connected by actin microfilaments and nexins, but maintain clear boundaries between individual cells.^62^ It is important to note that there exist small groups of 4–6 cartilage‐like NPCs in the NP with a diameter generally <10 μm, which must be distinguished from NCs. The distinction between NCs and cartilage‐like NPCs lies in the presence of vacuoles within the former's cells, which is absent in the latter.^34^ NCs are predominantly undifferentiated cells that exhibit a round, oval or polygonal morphology with a large volume and diameter ranging from 30 to 40 μm. These cells contain characteristic vacuoles, naive mitochondria, rough endoplasmic reticulum, cytoplasmic filaments and viscous intracytoplasmic glycogen.^63^, ^64^

As the most significant characteristic of NCs, vacuoles exhibit distinct morphological structures at different growth stages. Specifically, eNCs possess a complete and voluminous vesicle, whereas the vacuoles of NCs consist of multiple small and fragmented vesicles.^34^ Kim et al.^65^ discovered in their research that a single eNC, containing a complete large vacuole, could differentiate into a notochordal cell with numerous small and fragmented vacuoles within the cytoplasm. This finding further supports the notion that NCs and their vacuoles originate from eNCs.^66^, ^67^ Vacuoles are abundant in NCs, which are surrounded by dense actin microfilaments, accounting for ~56% of the cell volume as observed through actin staining of NCs.^64^

Confocal three‐dimensional reconstruction revealed that each notochordal cell contained an average of 7.2 vacuoles, with a volume of ~1870 μm^3^.^68^ Importantly, the vacuoles in vacuolated NCs play a crucial role in maintaining their morphology^47^ and are believed to be the driving force behind axial elongation during notochord development.^69^ The ion pump on the vacuole membrane generates a hypotonic solution in the vacuole, which can be released into the cytoplasm to dilute under osmotic pressure changes. This not only balances the osmotic pressure across the cell membrane,^70^ but also regulates cell volume and tension during rapid osmotic pressure changes, protecting NCs from damage caused by swelling and rupture.^71^ Therefore, the increase in vacuoles within NCs may indirectly indicate an elevation of pressure within the NCs. Additionally, these vacuoles are safeguarded by caveolae and a notochordal sheath rich in collagen, laminin, fibronectin and proteoglycans. This protection allows for buffering against sudden mechanical stress and rapid adaptation to acute mechanical stress.^72^, ^73^, ^74^

**FIGURE 1:**
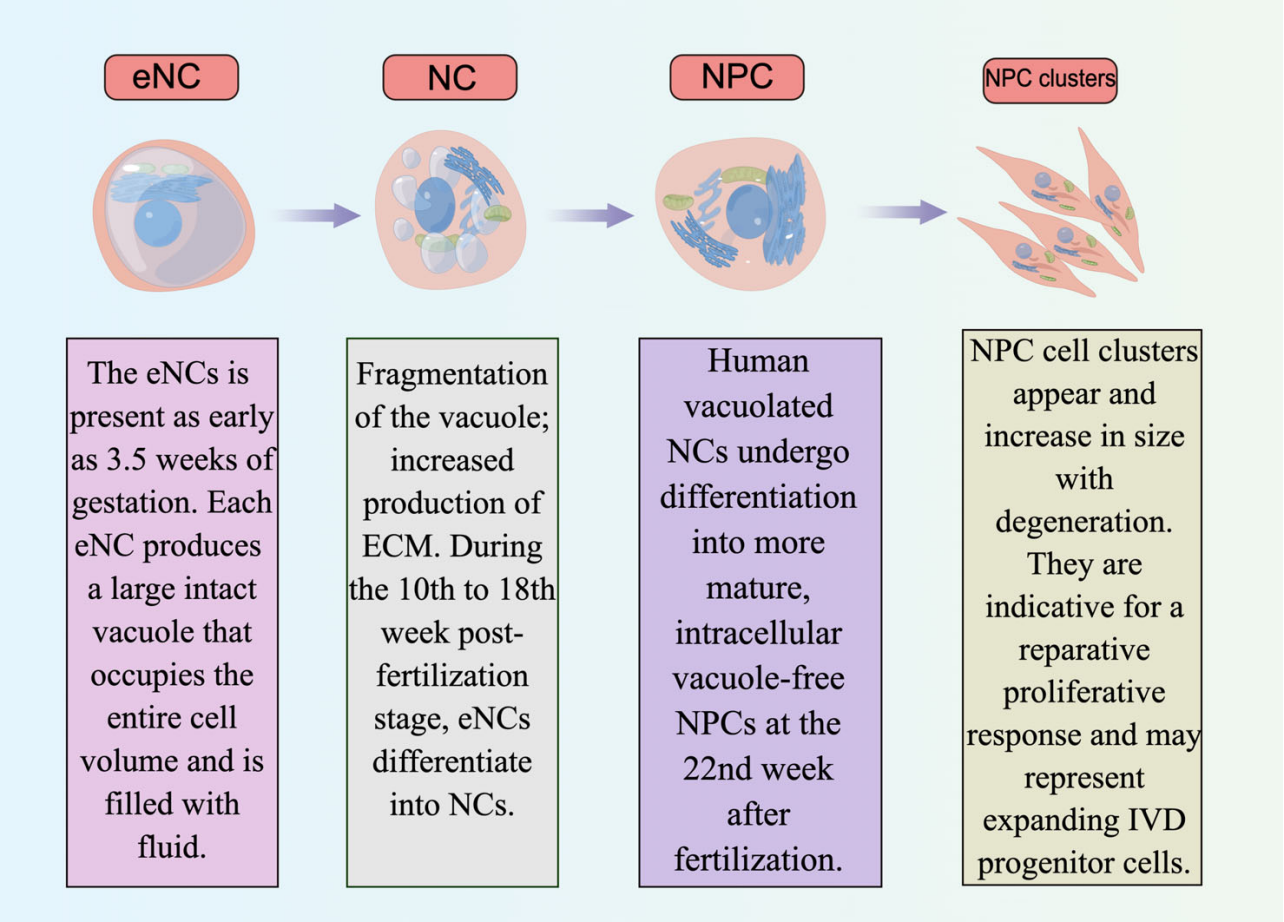
The biological process of notochordal cells (NCs) development into nucleus pulposus cells (NPCs) and its cell structure changes.

The mitochondria in NCs are relatively immature and are combined with the endoplasmic reticulum, which is a regular rectangular‐like structure with few or no folds, mostly located around the nucleus.^47^ This combination of immature mitochondria and rough endoplasmic reticulum suggests that the energy generated by vigorous anaerobic metabolism in NCs may be primarily utilised for protein synthesis. Due to the relatively limited energy supply of NP and the fact that glycogen reserves in cells mainly provide energy for NPCs, it is possible that the disappearance of NCs in adulthood is related to continuous glycogen consumption and insufficient energy supply, which may lead to cell death when glycogen is depleted.^75^ In addition, the cell membrane of notochords exhibits numerous villous bulges containing a small piece of eosinophilic cytoplasm in the centre. These bulges surround vacuole‐like nuclei with clear nuclei mostly located on one side and obvious perinuclear spaces.^47^ The biological process of NCs development into NPCs and its cell structure changes are shown in Figure 1.

### 2.3 Markers of NCs

Although the Spine Research Interest Group recommended a set of human NP markers, including Hypoxia‐inducible factor 1α (HIF‐1α), Sonic Hedgehog (SHH), Brachyury (T), Glucose transporter 1 (GLUT‐1), cytokeratin (CK) 18 and 19, Carbonic anhydrase 12 (CA12), CD24 and proteoglycan to type II collagen ratio >20 at the 2014 Annual ORS Meeting in New Orleans,^76^, ^77^ there have been limited studies on human notochordal cell markers thus far.^78^, ^79^, ^80^, ^81^ Previous studies have demonstrated significant differences in the expression of CK‐8,^82^, ^83^ CK‐18^84^ and CK‐19^85^ between NCs and NPCs in human NP tissue.^85^, ^86^, ^87^ These keratins can serve as reliable markers for distinguishing NCs from NPCs. The staining patterns of these three keratins are predominantly cytoplasmic with some perinuclear localisation, but not within vacuoles. Furthermore, their expression levels generally decline with NCs degeneration and ageing.^88^ This is highly consistent with the research findings of Rodrigues‐Pinto et al.^41^ who discovered that CK‐8, CK‐18 and CK‐19 are expressed in NCs throughout all stages of human development, thus suggesting their potential as specific markers for human NCs. Furthermore, they also observed CD24 expression in all developmental stages of human NCs after 3.5 weeks post‐conception, indicating its potential as a specific marker for human NCs.^41^

More importantly, it has been discovered that subsets of CD24‐positive mouse embryoid cells can spontaneously differentiate into cells exhibiting notochordal cell characteristics,^89^ providing further evidence to support the conclusion that CD24 is a specific marker for NCs.^90^, ^91^, ^92^ Furthermore, eNCs express CD55 specifically during the 7th to 9th week after fertilisation, making it a useful marker for identifying these cells.^41^ Therefore, CK‐8, CK‐18, CK‐19, CD24 and CD55 are viable positive markers for identifying NCs. However, E‐Cadherin (E‐Cad), which is present in chordoma cells, is not expressed during human NCs development.^93^ Similarly, tyrosine kinase receptor 2 (Tie2) and CD90 are not expressed at any stage of human NCs development, thus making them useful negative markers for identifying human NCs.^41^

Although the Brachyury (T) gene is involved in notochord formation during embryonic development,^94^ its aberrant expression can result in ectopic notochord formation, thus making it a useful marker for identifying notochordal cell phenotype.^95^, ^96^ However, the expression of Brachyury (T) is not exclusive to NCs. During the developmental stage from 5.5 to 18 weeks after fertilisation, sclerosed AF anlagen cells also express this marker, rendering it unsuitable as a specific indicator for NCs.^41^ Galectin 3 (GAL3) is a member of the galectin binding protein group, which interacts with various peptides and participates in diverse intracellular and extracellular signalling processes. These include regulating cell adhesion, RNA splicing and transducing extracellular signals. Some scholars suggest that GAL3 is expressed on NCs' surface, involved in glycation recognition, and can serve as a surface marker for NCs.^97^ However, Oguz et al.^98^ held a contrasting perspective by reporting the widespread expression of GAL3 in rat IVD and contending that this protein was not an appropriate marker for NCs.

Additionally, through the use of vimentin and connexin 43 antibody staining, researchers have identified both types of opals in NCs. Vimentin is primarily located within the cytoplasm,^99^ while connexin 43 is dispersed throughout the cell surface and concentrated at junctions between cells. Therefore, vimentin and connexin 43 can serve as phenotypic markers for NCs.^100^, ^101^ Moreover, Chen et al.^102^ utilised integrin α1, α6 and β1 antibodies for immunofluorescence staining of NCs and observed high expression levels of α1, α6 and β1 in NCs. Therefore, these markers can be employed to identify the phenotypic characteristics of NCs. Additionally, ganglioside 2,^103^, ^104^ as well as chondroid markers such as SOX9 and SOX5 expressed by NCs may also serve as indicators of NCs phenotype.^105^ Aquaporin‐transmembrane channel proteins have been proposed as potential markers for vacuoles of NCs, but further validation on human tissues is required.^106^

Although some progress has been made in the study of NCs markers, no specific markers have yet been identified that can exclusively label NCs at any developmental stage.^34^ Therefore, identifying such markers is a crucial direction for future research to elucidate the role of NCs in IVD development, maturation, degeneration and regeneration.

### 2.4 Solation and culture of NCs

#### 2.4.1 Separation methods of NCs and its disadvantages

Percoll density gradient centrifugation is the primary method for extracting NCs due to their lower density compared to cartilage‐like NPCs.^33^ First, NP tissues from young animals' thoracic and lumbar vertebrae were digested with type II collagenase. The resulting cell suspension was then filtered and centrifuged, collecting the precipitate from the centrifuge. After adjusting the cell suspension density, it was layered onto a discontinuous Percoll gradient for centrifugation. Following centrifugation, NCs were found in the upper layer while cartilage‐like NPCs settled in the lower layer. However, this method has two limitations. First, it is unfeasible to achieve NCs with high purity (100%).^107^ Second, purified NCs obtained by Percoll density gradient centrifugation produce significantly less proteoglycan than unpurified ones. This implies that the purification process may induce phenotypic changes in the NCs.^108^, ^109^

Flow cytometry and purification of NCs can be achieved through a new fluorescence‐activated cell sorting (FACS) protocol that includes auto‐fluorescence and size analysis. This technique allows for simultaneous measurement of multiple physical characteristics, such as size, granularity and fluorescence intensity, in single cells based on their light scattering and diffracting properties as they pass through a beam of light. Chen et al.^102^ utilised a novel FACS protocol incorporating auto‐fluorescence and size analysis to isolate and purify NCs from primary NPCs of 2–3 months old, 250–300 g weight rats. FACS analysis revealed that NCs isolated by this method exhibited lower expression levels of type I collagen, biglycan, TIMP1, HSP70 and c‐Fos compared to small cartilage‐like NPCs obtained via FACS. However, the expression levels of decorin, lumican, multiple MMPs and IL‐1β remained unchanged. There are two advantages to utilising this protocol. First, it enables the attainment of NCs with a purity level of 100%. Second, it facilitates the investigation of phenotypic and metabolic discrepancies between NCs in human NP tissue and those in adult chordoma. This may play a crucial role in developing therapeutic strategies that promote tissue regeneration, regulate cell differentiation and identify altered cell phenotypes. The drawback of this approach lies in the low yield of purified NCs, ranging from 15% to 30%, which is deemed unacceptable. Consequently, this protocol is not suitable for large‐scale isolation of NCs.^102^

A serial sieve‐filtration procedure was employed by Kim et al.^65^ to extract NCs from the NP tissue of adult New Zealand white rabbits, which contains a higher number of NCs compared to juvenile rabbits due to its larger size and maturity.^58^ First, the NP tissues of adult New Zealand white rabbits were excised and rinsed with Hank balanced salt solution. Subsequently, the specimens were subjected to digestion in Ham's F‐12 culture medium supplemented with 1% penicillin/streptomycin (P/S), 5% foetal bovine serum and 0.2% streptomycin for 1 h. Subsequently, the cells were rinsed with Hank balanced salt solution and subjected to overnight incubation at 37°C in the presence of 0.025% collagenase P. Following centrifugation through a sterile nylon filter (70 μm aperture) at 2000 RPM for 5 min, the cells were suspended and cultured in F‐12 medium supplemented with 10% foetal bovine serum and 1% P/S under a CO_2_‐enriched atmosphere (5%). Notably, due to their delayed adhesion capacity until day 6,^108^ NCs can be effectively isolated from cartilage‐like NPCs by day 3. Due to the significant difference in size between NCs and cartilage‐like NPCs, with NCs typically having a diameter >15 μm, the Serial sieve filtration procedure utilising meshes of 40, 25 and 15 μm can be employed to isolate NCs from cartilage‐like NPCs. Alternatively, a single notochordal cell can be obtained through pancreatic enzyme digestion of the NCs group. The method yields NCs with a purity exceeding 98%, yet complete purification remains unattainable. The extraction methods of NCs and their advantages and disadvantages are shown in Table 1.

**TABLE 1:** Methods of extracting notochordal cells (NCs) and their advantages and disadvantages.

Methods	Advantage	Disadvantage	References
Percoll density gradient centrifugation	Abundant NCs	Low purity and Phenotypic change	Aguiar et al.^33^; Erwin et al.^107^; Poiraudeau et al.^108^; Cappello et al.^109^
Flow cytometry and purification	High purity	Low yield	Chen et al.^102^
Serial sieve‐filtration procedure	Abundant NCs	Low purity	Alini et al.^58^; Kim et al.^65^; Poiraudeau et al.^108^

#### 2.4.2 Culture conditions for NCs

For the first time, Mwale et al.^110^ conducted a comprehensive study on the surface interaction between NCs and culture materials, providing valuable insights for selecting appropriate cell culture media. The authors cultured NCs in varying concentrations of nitrogen‐rich serum medium (PPE:N), which contained up to 36% N‐doped plasma‐polymerised ethylene, and found that these cells could maintain viability for up to 2 weeks. They also discovered that the chemical composition of the medium's surface can impact the expression of specific functional protein genes in NCs.^110^ Furthermore, Mahmoud et al.^111^ investigated the correlation between diabetic rats and IDD and found a positive association between hyperglycaemia and IDD. Similarly, another study demonstrated that a high sugar environment in vitro could impede NCs proliferation while promoting apoptosis.^112^ This clearly demonstrates that a high‐sugar environment is not conducive to the growth of NCs, thus it is recommended to use a low‐sugar medium for their cultivation.^113^, ^114^

Due to the loss of vacuolated phenotype and NCs‐specific markers during in vitro expansion and culture, maintaining the notochordal cell phenotype in vitro poses a significant challenge for both cell therapy and basic physiological research applications. To tackle this challenge, recent research has endeavoured to optimise the culture conditions of NCs. It has been discovered that culturing NCs in a 3D environment,^115^, ^116^ hypoxic conditions (1%–5% O_2_),^113^, ^117^ soft substrate surfaces (e.g., laminin coatings) with a stiffness <720 Pa,^118^, ^119^, ^120^ ECM mimicry peptides,^121^ serum‐free and low‐glucose media^113^, ^114^ as well as high osmolality (increased from 300 to 400 mOsm/L),^49^ can better maintain their morphological phenotype. In conclusion, to advance NCs‐based technologies in the field of regenerative medicine, it is imperative that we direct our attention towards this currently unexplored yet pivotal area.

## 3 POSSIBLE MECHANISMS OF NCS INVOLVED IN IDD

NCs can promote ECM synthesis and regulate cellular activity in the NP tissue of IVD, thereby playing a crucial role in maintaining normal IVD functional homeostasis.^64^ Previous studies have established that the reduction or loss of NCs is closely associated with the onset of IDD,^54^, ^75^ which initiates with apoptosis of NCs within NP.^122^ However, the specific mechanism underlying gradual decline of NCs in vivo remains unclear and may be linked to several processes.

### 3.1 FAS mediated apoptosis of NCs

FAS ligand (FASL) is a transmembrane protein of the tumour necrosis factor family,^123^, ^124^ which belongs to type II. FAS (apolipoprotein‐1/CD95) induces apoptosis in FAS‐positive cells by binding with its ligand.^125^, ^126^, ^127^, ^128^ The process of FAS‐mediated apoptosis does not require RNA or protein synthesis, and even enucleated cells can undergo apoptosis through FAS activation.^126^

Apoptosis induced by FAS may play a pivotal role in the process of NCs apoptosis.^129^ Previous studies have suggested that autocrine or paracrine FAS‐mediated counterattack could be a potential mechanism for rat NCs apoptosis.^130^ Furthermore, other investigations have demonstrated significant expression of FAS and FASL‐related proteins and mRNA in degraded IVD tissues (with increased levels of apoptosis) compared to normal IVD.^131^ This is consistent with the findings of Inui et al.,^132^ who observed that FAS ligand positive staining was absent in rat eNCs at 14.5 days, present in some eNCs at 16.5 days, and strongly expressed in most eNCs by 18.5 days. The aforementioned observations fully indicate that the expression of FAS and FASL in NCs increases with age, and is significantly correlated with NCs apoptosis. This provides evidence supporting the conclusion that FAS mediates NCs apoptosis. However, more importantly, Kim et al.^130^ results demonstrate that the mitochondria‐apoptosome‐caspase 9 pathway rather than the DISC‐caspase 8 pathway plays a crucial role in rat NCs' FAS‐mediated apoptosis.

Second, Kim et al.^130^ also found that the proliferation level of rat NCs expressing FAS and FASL was the highest at the 4th week after birth, and significantly decreased at the 6th and 12th month, but the apoptosis level was exactly the opposite. This further implies that the inverse correlation between proliferation and apoptosis of NCs, which is regulated by the FAS–FASL system, may be associated with the disappearance of NCs in rat IVD, representing another mechanism underlying IDD. In addition, previous studies have suggested that the FAS–FASL system may serve as a potential molecular mechanism for maintaining immune function in IVD,^133^ and it may also play an important role in the formation and normal physiological activities of NCs.^134^, ^135^, ^136^ Therefore, future investigations should focus on elucidating the specific involvement of the FAS–FASL system in notochordal cell reduction leading to IDD.

### 3.2 Oxidative stress activates caspase leading to apoptosis in NCs

In a subsequent investigation, Kim et al.^137^ subjected cultured NCs to hydrogen peroxide in vitro and observed that oxidative stress could trigger the activation of related proteins caspase‐9 (intrinsic apoptosis pathway), caspase‐8 (extrinsic apoptosis pathway) and caspase‐3 (common pathway). Thus, the apoptosis of NCs was found to be increased under oxidative stress conditions. However, inhibition of caspase‐8 and caspase‐9 did not significantly affect this apoptosis, whereas inhibition of caspase‐3 resulted in a significant reduction in oxidative stress‐induced NCs apoptosis.

Furthermore, it is reassuring that Suhl et al.^138^ obtained similar results when comparing the apoptosis of rat NCs under normal serum conditions and serum‐free conditions. They observed an increase in the expression of nerve growth factor (NGF), p75 receptor and downstream molecules of the c‐Jun N‐terminal kinase (JNK) signalling pathway when NCs were cultured under serum‐free conditions. Thus, the activation of the related proteins caspase‐9, caspase‐8 and caspase‐3 leads to increased apoptosis in NCs. However, under serum‐free conditions, the expression of molecules downstream of the signalling pathway that are beneficial to the survival of NCs, such as tropomyosin related kinase A (TrkA) receptors, protein kinase B (PKB/Akt) and mitogen activated protein kinase, is decreased in contrast to their expression levels in serum‐containing culture. Suhl et al.^138^ propose that NGF regulates the apoptosis and survival of NCs by selectively binding to its p75 receptor and TrkA receptor, while inhibiting the activity of caspase‐8 and caspase‐9 can reduce the incidence of apoptosis. Therefore, oxidative stress inhibitors may be utilised to combat oxidative stress‐induced apoptosis in NCs, which is expected to delay IDD onset.^6^

### 3.3 The differentiation of NCs into NPCs leads to IDD

**FIGURE 2:**
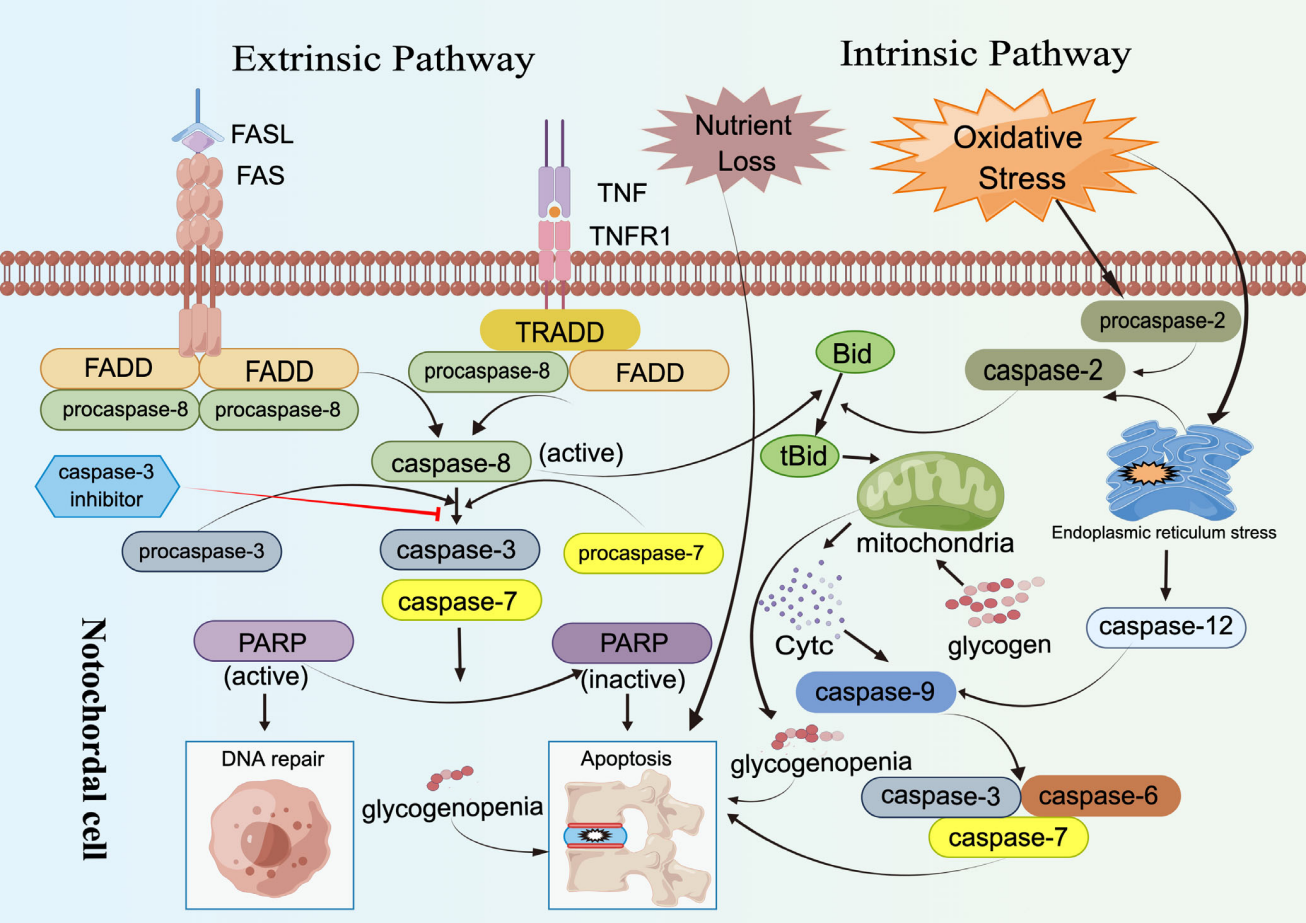
The possible mechanism by which notochordal cells (NCs) participate in intervertebral disc degeneration (IDD).

The differentiation of NPCs from NCs may contribute to IDD. In an acupuncture‐induced mouse model of IDD, NCs continuously differentiate into cartilage‐like NPCs and fibro chondroid cells, resulting in changes in cell phenotype and ECM composition. Specifically, the expression of type II collagen and proteoglycans decreases while the fibrocartilage phenotype marked by laminin and type I collagen increases, ultimately leading to a decrease in intervertebral disc height.^139^ Moreover, the loss of NCs in the IVD is also associated with decreased water content, increased collagen content and reduced proteoglycan content.^140^ Therefore, during continuous differentiation of NCs into cartilage‐like NPCs and final differentiation, cell phenotype changes accordingly. This affects normal matrix synthesis processes and cell metabolism in NP leading to IVD degeneration.^63^, ^139^ The possible mechanism by which NCs participate in IDD is shown in Figure 2.

## 4 NCS THERAPY FOR IDD

**FIGURE 3:**
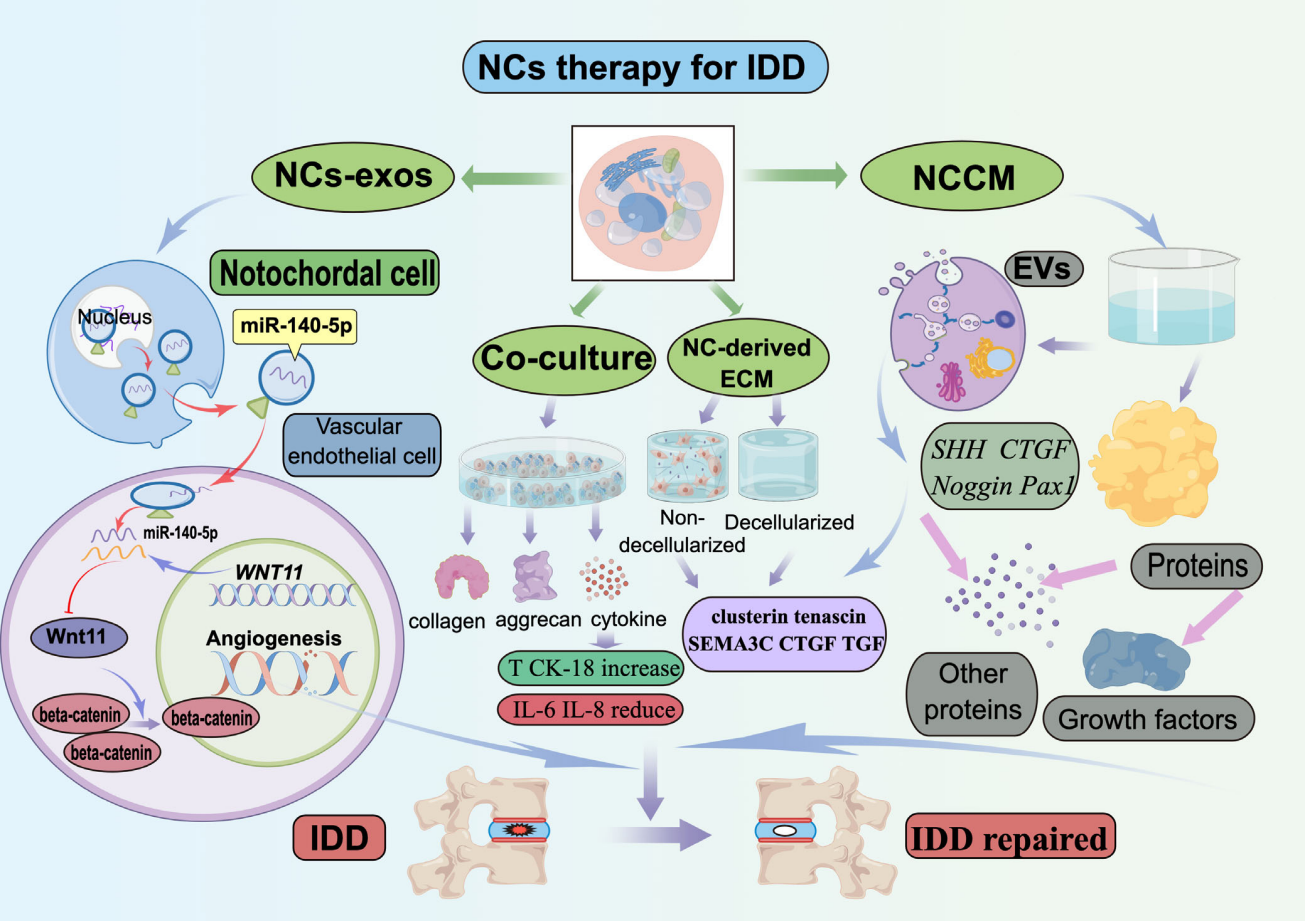
The four methods for treating intervertebral disc degeneration (IDD) based on notochordal cells (NCs).

Previous research has demonstrated that the retention of NCs exerts an inhibitory effect on inflammatory response, NPCs apoptosis and ECM degradation.^137^ Furthermore, NCs can enhance NPCs proliferation and their capacity to synthesise ECM. A significant positive correlation exists between the number of NCs and the ability of NPCs to generate ECM.^141^ The precise mechanism by which NCs facilitate NPCs‐mediated ECM synthesis remains elusive, with two main perspectives: “producer” and “director.” Cappello et al.^109^ proposed that NCs uniquely produce and converge ECM, acting as “producers” of aggrecan themselves. However, Erwin posited that NCs function as “conductors” in the synthesis of NP‐ECM, and the ECM secreted by NCs can induce MSCs to differentiate into NPCs expressing aggrecan and type II collagen. This provides a basis for selecting NCs therapy for IDD.^142^, ^143^, ^144^, ^145^ Therefore, given the crucial role of NCs in IVD biology, developing a NCs‐based treatment method for IDD holds far‐reaching significance and may offer fundamental therapeutic benefits. The four methods for treating IDD based on NCs are shown in Figure 3.

### 4.1 Therapeutic effect of co‐culture of NCs with other cells

Co‐culture studies typically involve the co‐cultivation of NCs with other types of stem cells or NPCs, which can synergistically contribute to a cell‐to‐cell signalling cascade through direct contact or the secretion of bioactive factors. Co‐culture experiments have demonstrated that NCs not only promote the proliferation and differentiation of bone marrow MSCs into NPCs, but also enhance the expression of type II collagen and aggrecan, as well as facilitate the production of a rich IVD matrix by MSCs, thereby promoting IDD repair.^146^

Similarly, Aguiar et al.^33^ were the first to observe an increase in aggrecan content in the system following co‐culture of NCs and NPCs. However, purified NCs exhibited minimal synthesis of aggrecan, leading to speculation that soluble cytokines secreted by NCs may promote its synthesis in NPCs. Therefore, it is crucial to focus on the investigation of soluble cytokines secreted by NCs in order to promote aggrecan synthesis in NPCs for a more profound treatment of IDD. Moreover, several studies have demonstrated that pig and dog NCs can enhance ECM production in bovine NPCs, indicating that the therapeutic effect of co‐cultured NCs with other cells is not species‐specific.^147^, ^148^

However, some studies have failed to observe this outcome, possibly due to alterations in the phenotype of NCs during prolonged culture under unfavourable physiological conditions.^149^, ^150^, ^151^ In addition, Kim et al.^152^ discovered that co‐culture with human macrophages and NPCs resulted in reduced expression levels of interleukin 6 (IL‐6) and interleukin 8 (IL‐8) genes and proteins in rabbit NCs, indicating that NCs can facilitate the repair of degenerative IVD by suppressing inflammatory response. More importantly, recent studies have demonstrated that co‐culturing rabbit NP tissue rich in NCs with human NPCs leads to increased proliferation of human NPCs, enhanced production of ECM and elevated expression levels of Brachyury (T) and CK‐18. These findings suggest that the co‐culture of NCs with other cells does indeed possess the ability to repair degraded IVD.^153^ Although co‐culturing NCs with other cells has demonstrated therapeutic benefits in degenerative IVD disease, there are several challenging issues that require resolution in future research. These include maintaining a stable phenotype of the NCs, addressing ethical concerns associated with this method, and managing the risk of tumour formation.

### 4.2 Therapeutic effect of conditioned culture medium for NCs

Conditioned culture medium refers to a type of culture medium that contains bioactive molecules secreted by cells during tissue or cell culturing, among which cytokines are one of the major components. The utilisation of conditioned culture media can effectively enhance the success rate of in vitro cell or tissue cultivation, and diverse origins of such media exhibit distinct promotional or inhibitory effects on various cells and tissues.

Notochordal cell‐conditioned medium (NCCM) shows promise as an alternative to co‐culturing for IDD‐related diseases. The preparation process involves culturing NP rich in NCs in a culture medium for 4 days, followed by collection of the conditioned medium through centrifugation and auxiliary filtration.^46^ Aguiar et al.^33^ have concluded that the incorporation of GAGs into NPCs by NCs is not solely dependent on cell–cell contact, but NCCM can also serve as a medium to facilitate this process. Since then, numerous studies have demonstrated that NCCM derived from various species (primarily pigs and non‐chondrodystrophic dogs) exhibit advantageous effects on NPCs,^154^, ^155^, ^156^, ^157^ MSCs,^158^, ^159^ AF cells^148^, ^160^ and CEP cells^161^, ^162^ under diverse conditions and culture media. The same experimental results indicate that NCCM can exert positive effects on the NP in degenerated IVD of both dogs^155^, ^156^ and humans,^163^ including enhancing ECM synthesis,^163^, ^164^ promoting cell proliferation^164^ and reducing cell apoptosis.^32^, ^157^

Additionally, the injection of NCCM into degraded IVD in rats has been shown to promote the regeneration of NPCs.^165^ Furthermore, the chondroitin sulphate found in NCCM can also inhibit axon and blood vessel growth,^143^, ^166^ thus demonstrating a beneficial effect on angiogenesis and neurogenesis inhibition.^167^ Due to its significant proliferative effect on NPCs and cytokine secretion promoting degenerative IVD repair, NCCM is considered a promising target for treating IDD. However, the precise mechanism by which NCCM exerts its nutritional function remains unclear and requires further elucidation. Recent research has indicated that NCCM may exert its effects through bioactive proteins secreted by NCs, which contain numerous proteins associated with the ECM, anti‐catabolic factors and growth factors, as well as Extracellular Vehicles (EVs).^159^, ^165^ Clusterin, tenascin and α2‐macroglobulin in NCCM are believed to confer protective effects against NPCs.^159^, ^168^ Matta et al.^165^ investigated NCCM in non‐chondrodystrophic dogs and found that ~31% of the identified proteins were associated with ECM. In addition, they identified multiple growth factors, including connective tissue growth factor (CTGF or CCN2), insulin‐like growth factor binding protein‐7, WNT‐induced soluble protein 2, TGF‐β and angiopoietin‐like 7.

CTGF secreted by NCs is a cysteine‐rich protein consisting of 349 amino acid residues with a molecular weight ranging from 36 to 38 kDa. It contains a thrombospondin module that interacts with TGF‐β, matrix metalloproteinases (MMPs), integrins and ECM proteins. Consequently, the expression of aggrecan, multifunctional proteoglycan and hyaluronic acid synthase 2 genes in NPCs was promoted.^169^ Studies have demonstrated a dose‐dependent correlation between the expression of aggrecan gene and CTGF, whereby higher concentrations of CTGF result in increased expression of aggrecan gene.^107^ Furthermore, CTGF has been implicated in apoptosis^170^, ^171^ and can independently stimulate ECM synthesis.^172^

Through proteomic analysis on canines and swine, scientists have detected three categories of SEMA, which could potentially exhibit anti‐angiogenic as well as anti‐neurogenic properties.^143^, ^167^ The presence of SEMA within NCCM among these animals corroborates its suppressive function towards both neural and vascular development.^173^ SEMA3C plays a crucial role in both angiogenesis and innervation of human IDD, and its association with LBP has been established.^174^ In recent years, combination therapy involving bioactive factors has shown promising results in the treatment of IDD. The key to successful combination therapy lies in maximising the positive effects of each bioactive factor while minimising their potential adverse effects within the NP. A combination of TGF‐β1 and CTGF has been demonstrated to effectively restore healthy, NCs‐rich NP tissue in preclinical rat models of induced IDD.^165^ Similarly, the administration of bioactive factors into canine degenerative IVD resulted in increased IVD height, enhanced ECM protein expression and reduced inflammatory mediator expression.^175^ However, this study did not investigate the potential promotion of tissue fibrosis or tumour development.^176^, ^177^, ^178^

EVs, small lipid bilayer‐enclosed particles released by cells, have garnered significant attention as potential targets for regenerative therapies in the cartilage or intervertebral disc domains.^179^, ^180^ The presence of vesicle‐associated proteins in non‐chondrodystrophic canine, porcine and human NCs‐conditioned medium suggests that EVs play a role in the biological function of these cells.^156^, ^181^ EVs act as protective carriers of biologically active molecules, such as mRNA, miRNA, DNA, proteins and lipids. They play a crucial role in cellular signalling and can interact with cells.^180^ *SHH* is also a pivotal factor in the development of notochords and IVD. Different vesicular miRNAs and proteins contained in EVs have diverse effects on *SHH*.^182^ The EVs secreted by NCs are abundant in ECM molecules and deposited in the NP tissue as a GAG‐rich matrix, which presents challenges for detecting growth factors.^156^ Studies have demonstrated that EVs, secreted in large quantities by pig NCs, exhibit anabolic effects on degenerative NPCs in dogs and humans.^163^ However, further investigation is required to determine whether EVs can alleviate symptoms through inhibiting inflammation, vascularisation and neurogenesis, as well as catabolism.

### 4.3 Therapeutic effects of NCs‐derived ECM

The notochordal cell‐derived ECM (NCM) can be either decellularized or non‐decellularized, and contains bioactive factors secreted by NCs that can supplement the ECM components of NPCs. Additionally, NCM can delay IDD through the genes of NCs, such as *SHH*, *CTGF*, *Noggin* and *Pax1*.^183^, ^184^ In terms of clinical therapeutic efficacy, NCM can be deemed comparable to demineralised bone matrix which is widely utilised for bone regeneration.^185^ Both materials are capable of promoting tissue repair through the local release of growth factors.^186^ The injection of NCM into degenerative IVDs may represent a promising therapeutic approach for IVD disease, circumventing the ethical and regulatory concerns associated with intracellular NCs therapy and the identification of certain bioactive NCs secretions.

Acellular ECM derived from porcine NCs has been shown to promote the proliferation of porcine synovium‐derived MSCs and induce the production of chondrogenic ECM, indicating that this microenvironment is conducive to differentiation into a NPCs‐like phenotype.^187^ Furthermore, in vitro studies have demonstrated that porcine NCM, which has similar protein concentrations as porcine NCCM, can increase both DNA and GAG content in bovine NPCs.^166^ Interestingly, when compared with NCCM from the same pigs, NCM was found to have a stronger anabolic effect on adult NPCs.^188^ In addition, NCM has anti‐inflammatory effects on bovine NPCs in vitro,^166^ and can improve local inflammatory responses in canine models of IDD in vivo.^186^

Although these results appear promising, the decellularisation of NCM remains a challenging obstacle to overcome in order to prevent host rejection of xenografts in clinical settings. However, studies on acellular porcine NCM have demonstrated that it can maintain human NPCs activity and enhance GAGs synthesis in vitro.^189^ In addition, human MSCs cultured on acellular porcine NCM express the NPCs marker and deposit GAGs and collagen.^190^, ^191^, ^192^, ^193^ The combination of acellular porcine NCM and rabbit MSCs can effectively treat the IDD model in rabbits and partially restore the ECM content of rabbit NPCs.^192^, ^193^ Xu et al.^192^ also detected abundant important signalling molecules, such as TGF‐β1, in the tissue structure of MSC‐NCM, which was consistent with protein results detected in native NCM.

It is noteworthy that cells possess numerous ligand‐binding domains crucial for cell‐ECM interactions, which are specifically linked to cytokines, chemokines, morpho factors and growth factors regulating cell differentiation and proliferation. Therefore, acellular components may impact the effects of ECM by influencing these bioactive factors.^194^ Additionally, the decellularisation process may have a detrimental effect on the GAGs and collagen content within the ECM of decellularized cells. Therefore, it is imperative to develop an optimal decellularisation protocol in order to maximise the biological functionality of NCM. Although porcine tissue‐derived products have been utilised in clinical applications,^195^, ^196^ further optimisation is required to ensure their safety while preserving biological activity. This includes the development of methods for removing genetic material^197^ and α‐Gal.^198^, ^199^ Acellular NCM can serve as a carrier during cell production to enhance the efficacy of cell therapy alone.

### 4.4 Therapeutic effects of NCs‐derived exosomes

Exosomes are extracellular vesicles in the 30–150 nm diameter range that are secreted by many cell types, including stem cells, tumour cells, immune cells and chondrocytes.^200^ They can transfer many bioactive substances, such as cytokines, proteins, lipids and RNA, and are often defined as intercellular communication vectors. Studies have shown that exosomes play an important role in tumour metastasis,^201^ immune regulation,^202^ osteoarthritis^203^ and angiogenesis.

Vascular invasion has been widely observed in IDD and is thought to play an important role in the progression of IDD by bringing about activated immune cells and inflammatory cytokines, facilitating neuralisation, and thus breaking the homeostasis of the IVD. There is growing evidence that NCs play an important role in IVD development and function.^204^ In particular, NCs not only activates NPCs^153^ and MSCs,^155^ but also stimulates cartilage differentiation and inhibits blood vessel growth by secreting a large number of biological factors.^143^ Thus, NCs has received considerable attention for its therapeutic potential. So, what exactly does NCs do to inhibit blood vessel growth? An important recent study showed that NCs‐derived exosomes (NCs‐exos) can be secreted by NCs and internalised by endothelial cells, miR‐140‐5p can inhibit angiogenesis by delivering NCs‐exos to endothelial cells with 0.5 MPa compression load induction and modulating the downstream Wnt/β‐catenin pathway, resulting in an avascular state of IVD.^205^ Moreover, the expression level of exosomal miR‐140‐5p in NP tissue was negatively correlated with angiogenesis in IDD.^205^ More importantly, results from animal models of IDD showed that 0.5 MPa/NCs‐exos reduced vascularisation of degenerative IVD tissue, and the progression of IDD was mitigated by micro‐CT measurement.^205^ This strongly suggests that NCs‐exos may be a potential option for further treatment of IDD.

Exosome‐derived miRNA can be transferred from various cell types to endothelial cells and exert an effective silencing effect on mRNA to reprogram the transcriptome. Numerous studies have shown that exosome‐mediated miRNA transfer to vascular epithelial cells is involved in angiogenesis regulation.^206^, ^207^ miR‐140‐5p has been shown to be involved in cell migration, proliferation and metastasis.^208^, ^209^, ^210^ Meanwhile, Wnt signalling pathway is closely related to angiogenesis by regulating endothelial cell proliferation, migration, vascular sprouting and vascular system maturation.^211^, ^212^ Importantly, β‐catenin regulation is considered to be one of the most important downstream pathways of Wnt‐dependent signalling.^213^, ^214^ miR‐140‐5p can activate β‐catenin to angiogenesis by inhibiting Wnt11.^215^, ^216^ In addition, the current study provides evidence that NCs‐derived miR‐140‐5p inhibits the secretion of MMP‐2 and MMP‐7 in endothelial cells.^205^ MMPs is a family of zinc‐containing and zinc‐dependent enzymes that have been shown to promote vascular infiltration by inducing degradation of the extracellular matrix (ECM).^217^ These results suggest that the exosomal miR‐140‐5p can reduce vascular invasion by inhibiting the expression of MMP. Although the role of NCs or NCs‐derived matrix in angiogenesis is still debated,^188^ studies have shown that NCs‐derived factors can inhibit angiogenic processes, indicating their potential role in inhibiting vascular in‐growth treatment.^143^

Recent studies have revealed novel therapeutic effects of exosomes derived from MSCs.^218^ In IVD, MSCs‐derived exosomes have been shown to treat IDD by transferring various biological factors and modulating corresponding downstream pathways.^219^, ^220^, ^221^ However, there is a lack of information in the literature on the existence of NCs‐exos and its effect on IDD. Therefore, future studies should pay more attention to the research of NCs‐exos, aiming to deepen the understanding of NCs‐exos in the treatment of IDD and develop new therapies based on NCs for the treatment of IDD.

## 5 CONCLUSION AND PROSPECT

This article provides a comprehensive review of the latest research progress on NCs, covering their biological characteristics, specific markers, possible mechanisms involved in IDD and its therapeutic effects. It also summarises significant future directions in this field. In conclusion, NCs and their secretions have been shown to promote NPCs proliferation, inhibit NPCs apoptosis and ECM degradation, suppress inflammatory response, and induce peripheral MSCs differentiation into NPCs for IVD repair. Given the pivotal role of NCs, this article primarily assesses their potential in managing LBP caused by IDD, while also outlining crucial future directions in this domain. This will aid in unravelling the pathogenesis of IDD and devising novel therapies for IDD based on NCs‐based therapeutic strategies.

Although the study of NCs holds promise for discovering novel approaches to treat IDD, safety concerns also exist, such as the formation of chordomas that contain cells with morphological similarities to NCs and express genes related to NCs.^222^ However, it remains unclear which priming mechanisms are essential for chordoma development. Fortunately, previous studies have demonstrated the presence of dormant NCs in adult vertebral bodies that, if activated, may proliferate and differentiate into chordoma, this occurrence is exceedingly rare.^223^, ^224^ And despite ~20% of adult vertebrae containing remnants of NCs, they do not typically progress to form chordomas.^225^, ^226^ Likewise, the incidence of chordomas in dogs is extremely low, with only two histologically confirmed cases reported so far, which may account for their rarity.^227^, ^228^ However, careful consideration of factors related to tumorigenesis is necessary when utilising regenerative NCs for IDD treatment. And it should be noted that research on NCs remains primarily in the animal experimentation stage. In addition to this, it has been confirmed that the soluble factor secreted by NCs can upregulate proteoglycan expression and promote MSCs differentiation into NPCs, making it a promising component of artificial ECM in tissue engineering repair systems. Therefore, patients suffering from IDD‐related pain may benefit from NCs‐based regeneration therapy in the future.^229^

Moreover, the articular cartilage of patients with osteoarthritis undergoes processes similar to those observed in advanced IDD, such as calcification and hypertrophic differentiation.^230^, ^231^ Given that NPCs share many similarities with chondrocytes and that NCs can stimulate NPCs regeneration, it is reasonable to hypothesise that NCs may also have a positive impact on chondrocytes.^162^ Similarly, ECM derived from NCs exhibits anabolic and anti‐inflammatory effects on healthy bovine articular chondrocytes. Additionally, it demonstrates lubrication properties that are comparable to hyaluronic acid.^232^ These findings suggest that substances secreted by NCs may have therapeutic potential not only for IVD disease but also for osteoarthritis and other orthopaedic conditions.^233^ To date, there have been no reports on the interaction between soluble cytokine extracts secreted by animal NCs and human NPCs.

Finally, the direct interaction between NCs and NPCs, as well as the potential direct or indirect interaction between NCs and other cells (such as MSCs), along with their specific modes of action, remain unclear. Further investigation into these issues will facilitate the development of superior tissue‐engineered IVD. By focusing on NCs in our research, we can open up a new avenue for treating IDD.

## AUTHOR CONTRIBUTIONS

Yanhu Li, Haijun Zhang, Daxue Zhu and Fengguang Yang put on the reference collection, reference analysis and manuscript writing. Zhaoheng Wang, Ziyan Wei, Zhili Yang and Jingwen Jia contributed to the topic conception. Xuewen Kang, the corresponding author, contributed to revising the manuscript and figures and decided to submit them for publication.

## FUNDING INFORMATION

This work was supported by the National Natural Science Foundation of China (82272536), Natural Science Foundation of Gansu Province (23JRRA1012) and (21CX1RA176).

## CONFLICT OF INTEREST STATEMENT

The authors declare that there are no conflicts of interest.

## Data Availability

Data sharing not applicable to this article as no data sets were generated or analyzed during the current study.
